# Immunogenicity of *Pv*Vir14-derived peptides to improve the serological diagnosis of *Plasmodium vivax* infection

**DOI:** 10.3389/fcimb.2025.1484863

**Published:** 2025-03-31

**Authors:** Ramayana Morais de Medeiros Brito, Raianna F. Fantin, Ana Laura Grossi de Oliveira, Ana Rafaela Antunes Porto, Isabela de Brito Duval, José Bryan da Rocha Rihs, Lilian Maria Lapa Montenegro Pimentel, Renata Maria Costa Souza, Elainne Christine de Souza Gomes, Joseli de Oliveira Ferreira, Daniella Castanheira Batholomeu, Ricardo Toshio Fujiwara, Lilian Lacerda Bueno

**Affiliations:** ^1^ Department of Parasitology, Institute of Biological Sciences, Federal University of Minas Gerais, Belo Horizonte, Brazil; ^2^ Laboratory of Immunobiology and Control of Parasites, Institute of Biological Sciences, Federal University of Minas Gerais, Belo Horizonte, Brazil; ^3^ Department of Microbiology, Icahn School of Medicine at Mount Sinai, New York, NY, United States; ^4^ Center for Vaccine Research and Pandemic Preparedness, Icahn School of Medicine at Mount Sinai, New York, NY, United States; ^5^ Department of Immunology, Laboratory of Immunoepidemiology of Aggeu Magalhães Institute, Oswaldo Cruz Foundation, Recife, Pernambuco, Brazil; ^6^ Department of Parasitology, Aggeu Magalhães Institute, Oswaldo Cruz Foundation, Recife, Pernambuco, Brazil; ^7^ Laboratory of Immunoparasitology, FIOCRUZ Foundation, Recife, Pernambuco, Brazil

**Keywords:** Malaria, *Plasmodium vivax*, PvVir14, immunogenicity, diagnostics

## Abstract

**Introduction:**

Malaria, caused by *Plasmodium vivax*, remains a major global health problem, particularly in tropical and subtropical regions. This study aimed to investigate the potential of *Pv*Vir14-derived peptides to improve diagnostic accuracy for *P. vivax* infections.

**Methods:**

The reactivity of specific *Pv*Vir14-derived peptides against sera from acutely infected individuals from endemic and non-endemic areas experiencing a *P. vivax* malaria outbreak was assessed, as well as their sensitivity, specificity, and immunodominance.

**Results:**

Among the eight tested peptides (H08, K09, I03, G08, F08, H04, L06, and N04), H08, G08, and L06 showed significantly higher IgG reactivity in sera from individuals living in endemic areas, mainly within those with multiple malaria episodes. After a *P. vivax* outbreak in a non-endemic area, H08 and G08 had the highest IgM frequencies, however, sensitivity and specificity analyses indicated that L06 was the most effective peptide for identifying infected individuals. Depletion ELISA confirmed the immunodominance of L06, G08, and H08 and showed a significant reduction in IgG reactivity to *Pv*Vir14. The peptides L06, G08, and H08, showed high sensitivity and specificity as diagnostic tools for *P. vivax* malaria.

**Conclusions:**

These peptides can improve diagnostic accuracy, especially in endemic areas, providing better support to malaria control and management programs.

## 1 Introduction

Malaria, caused by parasites of the genus *Plasmodium*, is one of the most globally impactful protozoan diseases. Among the species that affect humans, *Plasmodium falciparum* and *Plasmodium vivax* are the most prevalent (WHO, 2023).

Differential diagnosis between malaria caused by *P. vivax* and *P. falciparum* is crucial for effective patient management, as it ensures the correct treatment choice and helps to avoid disease exacerbation, unnecessary treatments, and drug resistance (Tjitra et al., 2008; Price et al., 2020).

The VIR superfamily of *P. vivax* comprises surface molecules that are associated with virulence and evasion in this parasite species (Fernandez-Becerra et al., 2009; Bernabeu et al., 2012). Proteins from this group are exported to the membrane of infected reticulocytes, facilitating their presentation to the immune system and the production of antibodies that can be used as exposure markers in human populations (Oliveira et al., 2006; Bernabeu et al., 2012). Here, we identified, selected, and evaluated the reactivity of peptides derived from *Pv*Vir14, a specific protein that circulates during acute *P. vivax* infection (Fantin et al., 2022).

## 2 Materials and Methods

### 2.1 Study population and ethical statement

The study enrolled 135 individuals from the endemic area of *Plasmodium vivax* malaria in Porto Velho, Rondônia State in the Amazon region of Brazil. Of these, 117 individuals had their infection diagnosis confirmed by positive blood smears, while 18 individuals did not have acute malaria and were considered exposed. Additionally, 153 individuals residing in a non-endemic area for malaria in the municipality of Conde, Paraíba state, Northeastern Brazil, who had experienced an outbreak of *P. vivax* malaria, were included. Of the total number, 17 individuals had their malaria diagnosis confirmed by blood smears, while 136 individuals did not have the infection and were considered exposed. The blood samples from the individuals affected by the outbreak were collected in July of 2019. As a negative control, sera from 15 healthy individuals living in non-endemic areas of the Brazilian state of Minas Gerais were collected. All participants were over 18 years of age and provided informed consent to participate in the study.

This study was conducted following the ethical standards of the Research Ethics Committee of the Oswaldo Cruz Foundation (FIOCRUZ) in Pernambuco, and by the Universidade Federal de Minas Gerais (UFMG), Brazil. The study was approved by the Research Ethics Committee of UFMG under protocol CAAE: 27466214.0.0000.5149, and by the Research Ethics Committee of the FIOCRUZ under protocol CAAE: 15891619.1.0000.5190.

### 2.2 Peptides *SPOT* synthesis, immunoblotting and soluble peptide synthesis

Peptide arrays corresponding to the entire protein *Pv*VIR14 were synthesized on a nitrocellulose membrane, as previously described (Siqueira et al., 2023), and probed using a pool of serum from healthy subjects (Negative Control, NC), and in a second moment with serum from patients with active *P. vivax* (Positive Control, PC). Peptides that demonstrated reactivity in both the NC and PC groups were excluded from further analysis, according to the previously established methodology (Bueno et al., 2011; Fantin et al., 2021).

The immunoblotting assay was conducted as previously described (Siqueira et al., 2023), with minor alterations. In brief, the peptide’s ability to recognize specific IgG antibodies was evaluated using a pooled serum sample (dilution 1:500) from 10 individuals with active *P. vivax* infection (PC), residing in an endemic region. For detection, a secondary anti-human IgG antibody was applied at a 1:10.000 dilution. As NC, a pooled serum sample from 10 healthy individuals from non-endemic, malaria-free areas was utilized. The quantification of spot signal intensities was conducted using the ImageQuant LAS 4000 digital imaging system software, with the analyses performed using ImageJ.

The densitometric value of each spot was determined and normalized using ImageJ, as previously described (Siqueira et al., 2023). After the membrane analysis, eight peptides were able to be synthesized in their soluble form using the solid-phase peptide synthesis (SPPS) technique on a 10 µmol scale using the ResPep SL automated synthesizer (Intavis) as previously described (Ruas et al., 2025). The eight successfully synthesized peptides then proceeded for subsequent immunogenicity analyses.

### 2.3 Quantification of total IgM and IgG

To determine the presence of specific IgG and IgM antibodies against *Pv*Vir14 and its derived peptides, an Enzyme-linked Immunosorbent Assay (ELISA) was performed as previously described (Fantin et al., 2023). This assay was carried out using serum from individuals from both endemic and non-endemic areas of *P. vivax* malaria. Sera from individuals residing in non-endemic areas and who had never experienced malaria were utilized as negative controls. The cut-off values were calculated as previously described (Fantin et al., 2021; Fantin et al., 2023), and the samples were considered positive when Reactivity Index (R.I.) ≥1 (Bueno et al., 2011).

### 2.4 Depletion ELISA

The depletion ELISA was performed to evaluate the influence of peptides (L06, H08. And G08) on the recognition of PvVir14 by specific IgG antibodies, following the methodology earlier described by (Santiago et al., 2011). In brief, 96-well plates were coated with 2 µg/well of each analyzed peptide and incubated overnight at 4°C. Subsequently, the plates were washed and blocked under the protocol. The diluted sera (1:100) were then added and incubated overnight at 37°C. On the following day, the sera were transferred to 96-well plates previously coated overnight with *Pv*Vir14 (0.5 µg/well) after washing and blocking. The ELISA was then conducted by the previously described methodology.

### 2.5 Statistical analysis

All data presented here was analyzed using GraphPad Prism 8.0 (GraphPad, San Diego, USA). The normality distribution of all data was analyzed by the Shapiro-Wilk test. Kruskall-Wallis followed by Dunn’s post-test was performed. Fisher’s exact test was used to determine the equality of variances among normal populations. The receiver operating characteristic (ROC) curve was employed to analyze the specificity and sensitivity of *Pv*Vir14 and derived peptides. All data were considered statistically significant when p < 0.05.

## 3 Results

### 3.1 Study population and selection of *Pv*Vir14-derived peptides

The study involved 135 participants from an endemic area of *P. vivax* malaria in Brazil, with a gender distribution of 31.86% female (n=43) and 68.14% male (n=92). The average age was 54 years (± 17.28) for females and 46 years (± 13.62) for males. Regarding previous malaria infections, 21.48% (n=29) had no history of malaria, 20% (n=27) had 1-2 previous episodes, and 58.52% (n=79) had three or more episodes (**Table 1**).

**Table 1:** General characteristics of the enrolled participants from *P. vivax* endemic area.

Age	Years (± SD)	
Female	54 (± 17.28)	
Male	46 (± 13.62)	
Gender	N	%
Female	43	31.86
Male	92	68.14
Total	135	100
Previous Malaria		
0	29	21.48
1	27	20
≥2	79	58.52
Total	135	100
Symptoms		
Fever	97	71.85
Headeache	99	73.33
Shivering	70	51.85
Nausea	38	28.15
Myalgia	72	53.33
Others	32	23.7

N, number of individuals; SD, Standard Deviation; % percentage value, considering the total sample size of 135 individuals.

**Figure 1:**
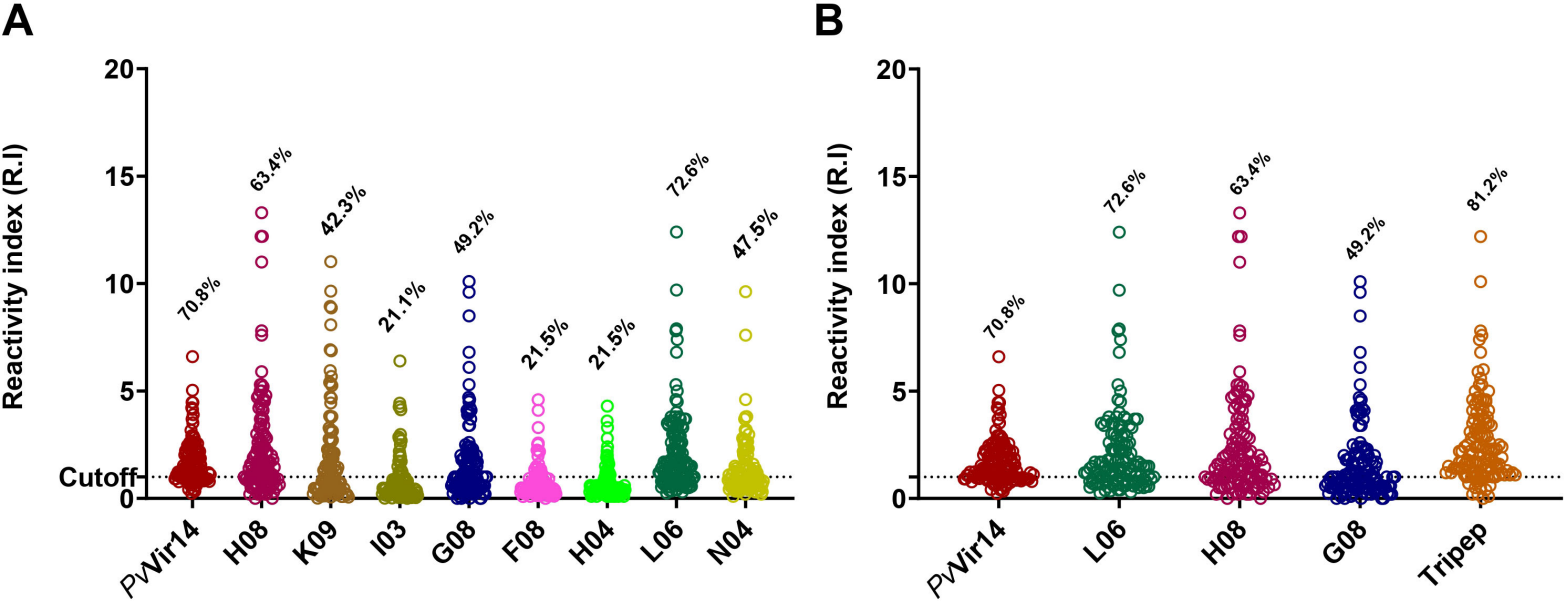
Densitometry analysis and reactivity frequency of PvVir14-derived peptides in individuals from *P. vivax* endemic area. **(A)** Reactivity frequency for specific IgG antibodies against the PvVir14 protein and the eight selected peptides in the serum of individuals from the *P. vivax* endemic area. **(B)** Reactivity frequency for specific IgG antibodies against the PvVir14, the three selected peptides (L06, H08 and G08), and the three peptides combined in the serum of individuals from the *P. vivax* endemic area. Samples were considered positive when reactivity index (R.I.) ≥ 1, which represents the cut-off value shown in the graphs by the horizontal dotted lines.

The densitometry results were analyzed (**Table 2**), and the eight peptides successfully synthesized (identified as H08, K09, I03, G08, F08, H04, L06, and N04) were found to be recognized by specific IgG antibodies present in the sera of individuals from endemic areas for *P. vivax* malaria. The frequency of individuals that are positive for the peptides H08, G08, and L06 was found to be significantly higher than that of the others (p<0.0001, Fisher’s exact test) (**Figure 1A**). It was observed that 70.8% of the individuals exhibited reactivity to the *Pv*Vir14 whole protein alone. Concerning the synthesized peptides, H08, G08, and L06 demonstrated a frequency of 63.4%, 49.2%, and 72.6% reactivity in the sera, respectively. When the three peptides were pooled together, the reactivity frequency increased to 81.2% (**Figure 1B**).

**Table 2:** Densitometric characteristics of *Pv*Vir14-derived peptides and their reaction against serum samples from non-infected and *P. vivax*-infected individuals.

Peptide ID	Molecular Weight (Da)	NC (AU)*	PC (AU)*	PC: NC ratio
F08	1203.53	2144.25	60211	28,080215
G08	1184.55	2526.25	64772	25,639584
H04	1124.61	2552.25	54295	21,273386
H08	1250.56	1418.25	70025	49,374229
I03	1048.46	1033.25	66371	64,23518
K09	954.42	5257.25	69349	13,191117
L06	1032.55	2540.25	44074	17,350261
N04	989.53	2797.25	40621	14,521762

NC, negative control (healthy individuals); PC, positive control (*P. vivax-infected* individuals). *Densitometric values are expressed in arbitrary units (AU), representing the relative intensity of spot signals.

### 3.2 *Pv*Vir14-derived peptides reactivity in endemic areas for Malaria

**Figure 2:**
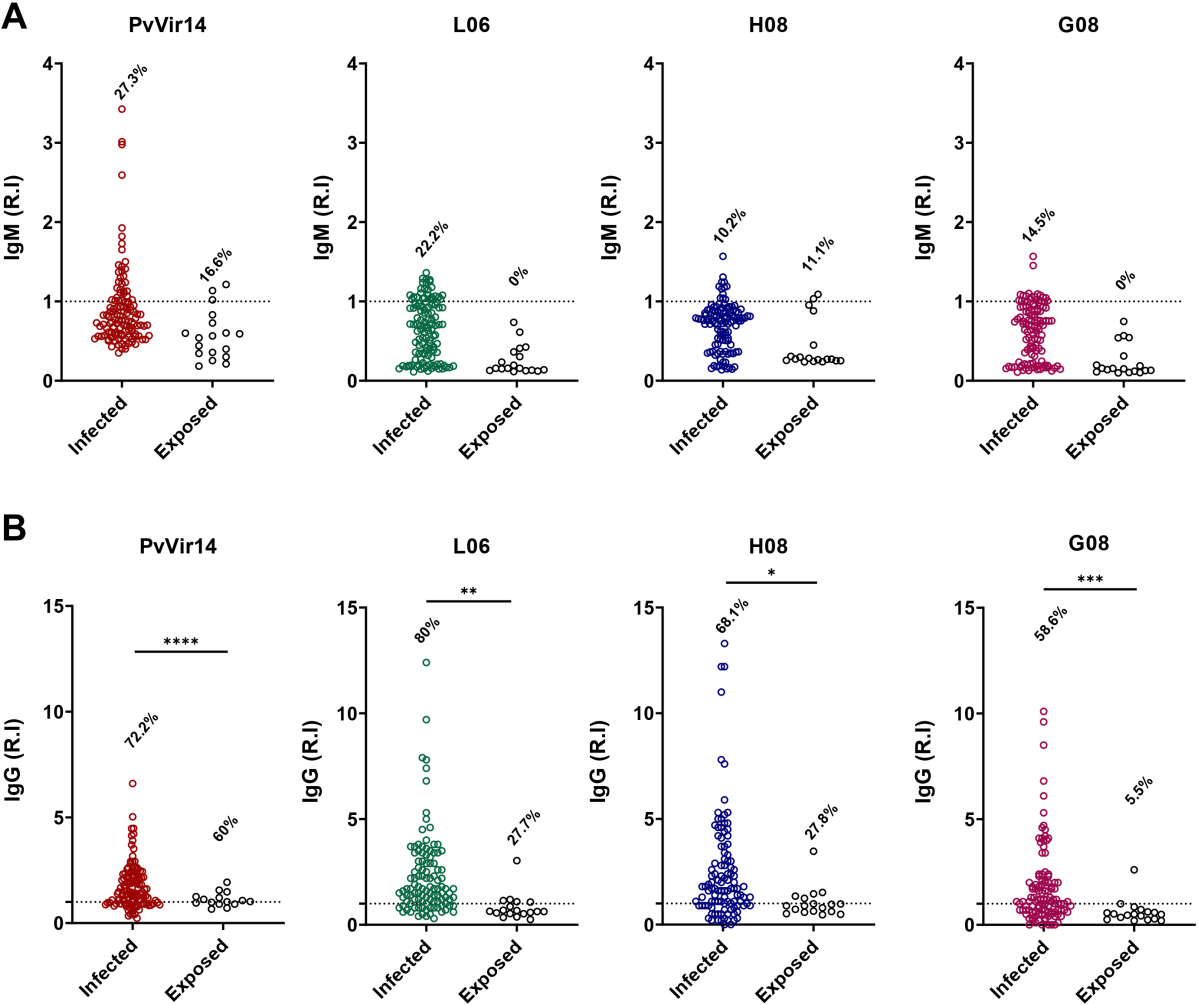
IgM and IgG antibodies reactivity frequency within individuals from endemic area for *P. vivax* malaria. The individuals were divided into those with acute *P. vivax* malaria (identified as infected), and those without acute infection (identified as exposed). **(A)** IgM antibodies reactivity frequency against PvVir14 protein, and the peptides L06, H08, and G08. **(B)** IgG antibodies reactivity frequency against PvVir14 protein, and the peptides L06, H08, and G08. Samples were considered positive when reactivity index (R.I.) ≥ 1, which represents the cut-off value. * p<0.05; ** p<0.01; *** p<0.001; **** p<0.0001.

After analyzing the frequency of IgG reactivity among individuals from the endemic area, we classified the population into two different categories: (i) with acute malaria infection and compared to (ii) non-infected but exposed to malaria participants. Specific IgM reactivity to *Pv*Vir14, L06, H08, and G08 was observed in 27.3%, 22.2%, 10.2%, and 14.5% of *P. vivax*-infected individuals, respectively (**Figure 2A**). The frequency of total IgG reactivity was higher among infected individuals, with 72.2% (p<0.0001), 80% (p=0.0025), 68.1% (p=0.0104), and 58.6% (p=0.0008) for *Pv*Vir14, L06, H08, and G08, respectively (**Figure 2B**).

**Figure 3:**
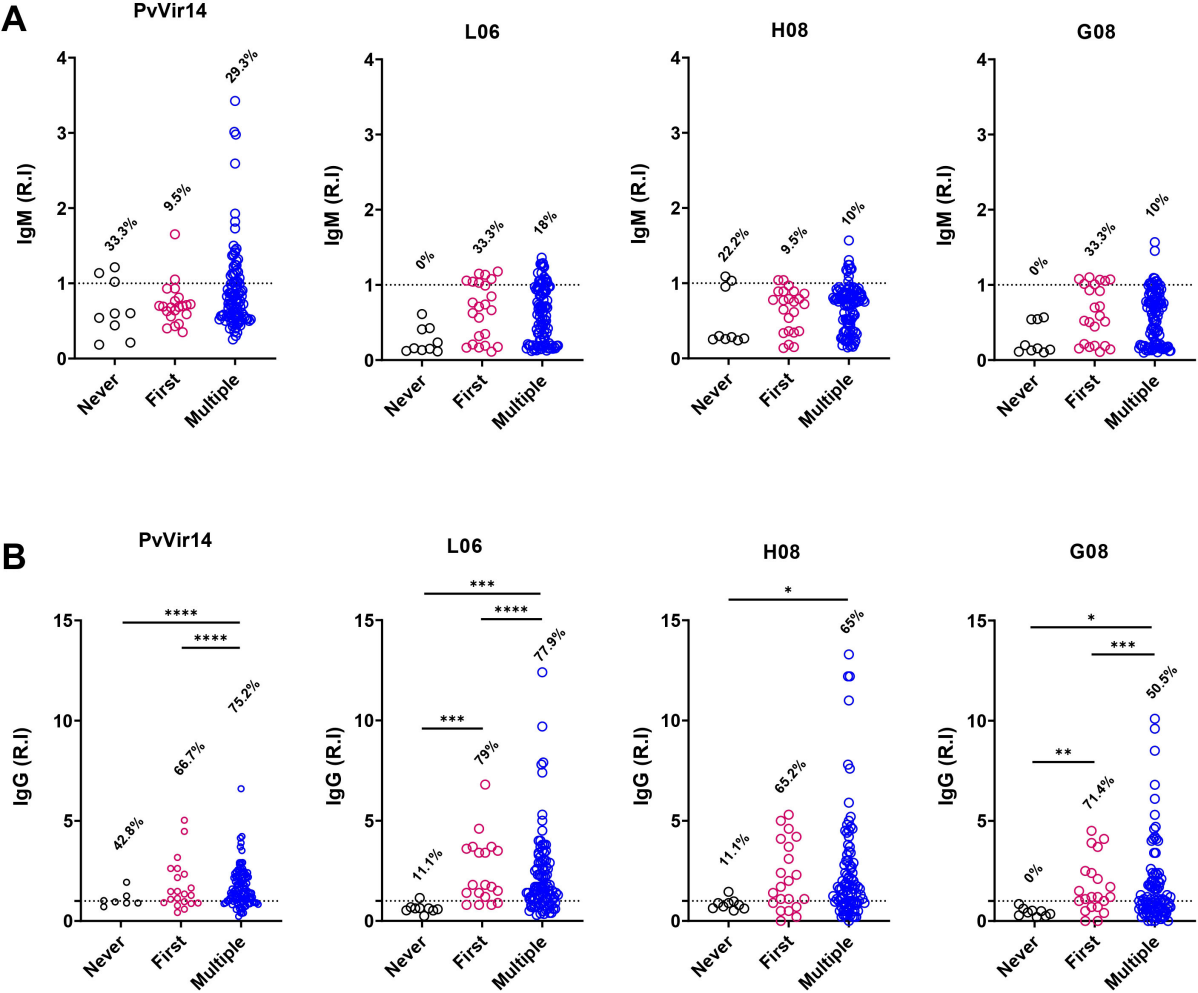
IgM and IgG antibodies reactivity frequency within individuals from endemic area, according to their malaria history. The individuals were divided according to their malaria history: those that never had malaria (black circles); those that were in their first malaria episode (pink circles); and those who suffered multiple malaria episodes (blue circles). **(A)** IgM antibodies reactivity frequency against PvVir14 protein, and the peptides L06, H08, and G08. **(B)** IgG antibodies reactivity frequency against PvVir14 protein, and the peptides L06, H08, and G08. Samples were considered positive when reactivity index (R.I.) ≥ 1, which represents the cut-off value.

The individuals from the endemic area enrolled in the study were then organized into three groups based on their malaria history: (1) Those who were exposed but never exhibited the acute infection, (2) those who experienced their first malaria episode, and (3) those who had two or more cases of malaria. The IgM reactivity for *Pv*Vir14 was 33.3% and 29.3% for the first and third groups, respectively (**Figure 3A**). Among individuals experiencing their first episode of malaria, the frequency of IgM reactivity was found to be 33.3% for the peptides L06 and G08, and 9.5% for the peptide H08 (**Figure 3A**).

The IgG reactivity against *Pv*Vir14 and the peptides was higher in individuals with the first or multiple cases of malaria (**Figure 3B**). Anti-*Pv*Vir14 IgG reactivity had a frequency of 75.2% in individuals with multiple cases of malaria, while those in the first episode of the disease displayed a frequency of 66.7% (p<0.0001, Fisher’s exact test). Individuals experiencing their first episode of malaria had higher IgG reactivity against the peptides L06 (79%, p<0.0001), and G08 (71.4%, p=0.0003), when compared to those with no previous malaria history, and those with multiple cases of infection. For individuals in their first malaria episode and those with multiple cases of malaria, the frequency of IgG reactivity to the peptide H08 was 65.2%, and 65%, respectively (**Figure 3B**).

### 3.3 Reactivity of *Pv*Vi r14-derived peptides in individuals from a malaria non-endemic region

Considering the 153 individuals from a non-endemic area for Malaria, who experienced the outbreak, 56.35% (100) were female with a median age of 35 years; the male individuals (34.65%) had a median age of 43 years. In terms of previous malaria episodes, 84.31% had no prior infections, 13.07% had experienced 1 to 2 previous infections, and 2.61% had experienced 3 or more previous infections. Additionally, 13.72% (21) of the individuals had previously visited a malaria-endemic area, while 86.27% (132) had not (**Table 3**).

**Table 3:** General characteristics of the enrolled participants from non-endemic area for malaria that experienced a *P. vivax* outbreak.

	N	%
Gender		
Female	100	56.35
Male	53	34.65
Total	153	100
Age (median ± SD)		
Female	35 (± 16.56)	–
Male	43 (± 19.91)	–
Education (in years)		
0 – 4	66	43.13
5 – 8	37	24.18
> 9	38	28.83
Total	141*	96.14*
Previous Malaria		
0	129	84.31
1 – 2	20	13.07
≥ 3	4	2.62
Total	153	100
Previous visit to Malaria endemic area		
Yes	21	13.72
No	132	86.28
Total	153	100
Symptoms		
Fever	12	7.84
Headache	38	24.83
Shivering	3	1.96
Nausea	2	1.3
Myalgia	2	1.3
Others	13	8.49

N, number of individuals; % percentage value, considering the total sample size of 153 individuals. *12 individuals did not report any education level.

**Figure 4:**
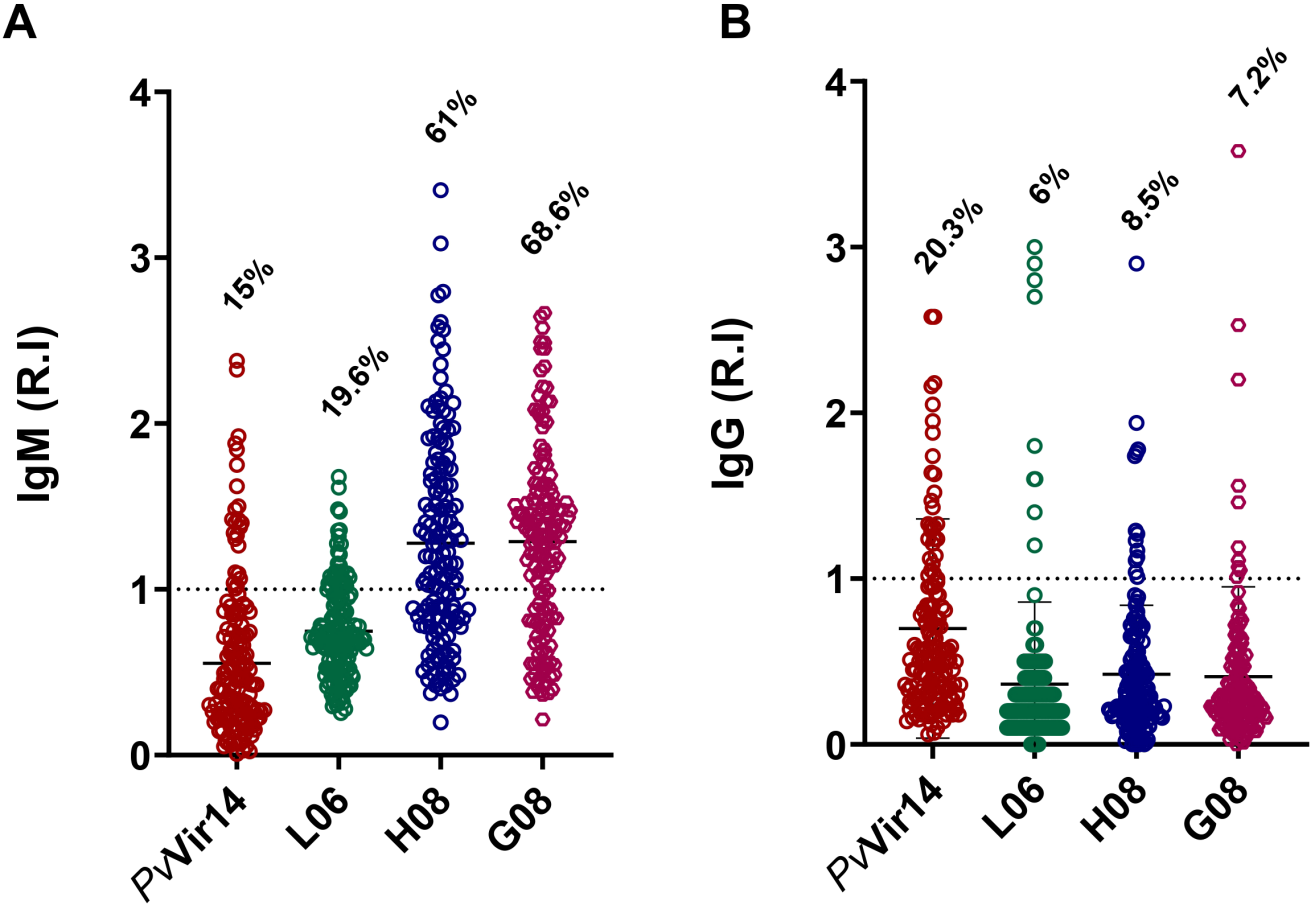
Seroreactivity of IgM and IgG antibodies against PvVir14 and derived peptides in individuals from a non-endemic area for Malaria, after *P. vivax* outbreak. **(A)** IgM antibodies reactivity frequency against PvVir14 protein, and the peptides L06, H08, and G08. **(B)** IgG antibodies reactivity frequency against PvVir14 protein, and the peptides L06, H08, and G08. Samples were considered positive when reactivity index (R.I.) ≥ 1, which represents the cut-off value.

**Figure 5:**
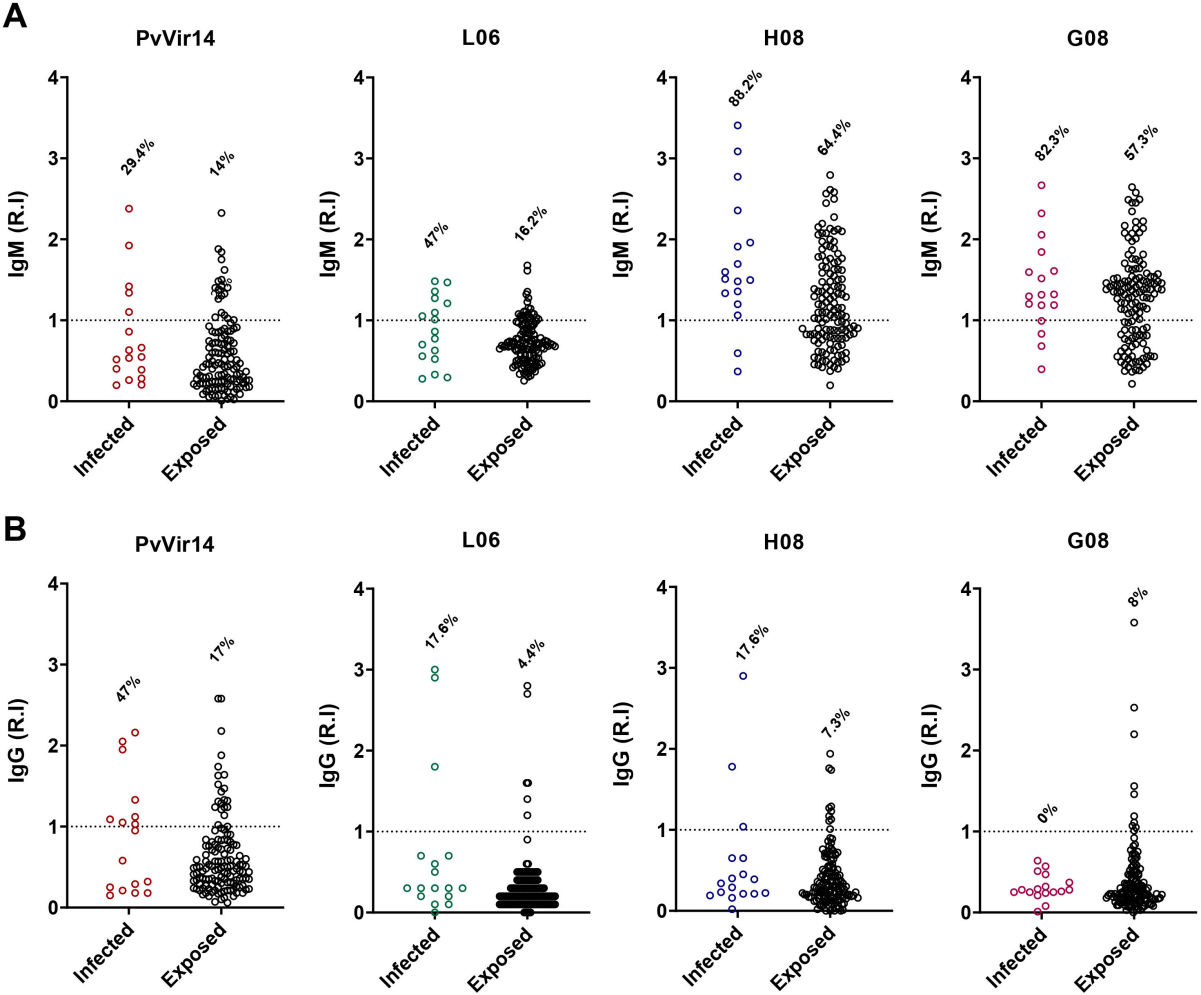
IgM and IgG antibodies reactivity frequency within individuals from non-endemic area for *P. vivax* malaria. The individuals from the *P. vivax* outbreak area were divided into those with acute *P. vivax* malaria (identified as infected), and those without acute infection (identified as exposed). **(A)** IgM antibodies reactivity frequency against PvVir14 protein, and the peptides L06, H08, and G08. **(B)** IgG antibodies reactivity frequency against PvVir14 protein, and the peptides L06, H08, and G08. Samples were considered positive when reactivity index (R.I.) ≥ 1, which represents the cut-off value.

The highest IgM reactivity was found for the peptides H08 (61%) and G08 (68.6%) (**Figure 4A**). Regarding the frequency of anti-*Pv*Vir14 IgG, 20.3% of the participants had circulating antibodies. In contrast, the frequency of antibodies for the three peptides was lower than 10% (**Figure 4B**). Within the individuals with acute infection, the frequency of IgM against *Pv*Vir14, and anti-L06, -H08, and -G08 was 29.4%, 47%, 88.2%, and 82.3%, respectively (**Figure 5A**). Specific anti-*Pv*Vir14 IgG reactivity presented a frequency of 47% within infected individuals, while IgG against the peptides L06 and H08 was equal (17.6%), for the peptide G08 displayed 0% of reactivity for the same group (**Figure 5B**).

### 3.4 Evaluation of sensitivity and specificity of *Pv*Vir14 and derived peptides

In the endemic area, peptides L06, H08, and G08 exhibited similar sensitivity and specificity values exceeding 80% when compared to *Pv*Vir14 values for infected individuals against healthy controls (**Supplementary Figure S1A**). Nevertheless, L06 was the most effective peptide for identifying infected individuals (**Table 4**). When analyzing the ability of the protein and peptides to differentiate infected individuals from exposed individuals, *Pv*Vir14 demonstrated sensitivity and specificity values exceeding 90% (**Table 4**). Only the peptide H08 was able to discriminate between infected individuals from exposed ones, with sensitivity and specificity values of 61.1% and 72.7%, respectively (**Supplementary Figure S1B**; **Table 4**).

**Table 4:** Sensitivity and Specificity of *Pv*Vir14 and the peptides L06, G08, and H08 tested in infected and exposed malaria patients, and healthy individuals.

Infected vs Healthy								
ID	Cut-off	AUC (± SD)	Sensitivity (%)	95% C.I. (%)	Specificity (%)	95% C.I. (%)	Likelihood ratio	*p*-value
*Pv*Vir14	0.2440	0.9449 (± 0.02)	90.91	84.45% - 94.85%	83.33	43.65% - 99.15%	5.455	*0.0002*
L06	0.1430	0.9236 (± 0.25)	88.7	81.62% - 93.27%	85.71	48.69% - 99.27%	6.209	*0.0002*
G08	0.1160	0.8271 (± 0.05)	80	65.24% - 89.50%	83.3	43.6% - 99.15%	4.800	*0.0105*
H08	0.066	0.8548 (± 0.03)	81.82	74% - 87.6%	85.71	48.69% - 99.27%	5.727	*0.0016*
Infected vs Exposed								
ID	Cut-off	AUC (± SD)	Sensitivity (%)	95% C.I. (%)	Specificity (%)	95% C.I. (%)	Likelihood ratio	p-value
*Pv*Vir14	0.2111	0.9620 (± 0.01)	93.33	70.18% - 99.66%	92.56	86.47% - 96.04%	12.55	*<0.0001*
L06	0.2851	0.5592 (± 0.06)	50.00	29.03% - 70.97%	61.74	52.61% - 70.11%	1.307	0.4204
G08	0.2273	0.5819 (± 0.07)	50.00	29.03% - 70.97%	57.50	42.20% - 71.49%	1.176	0.3214
H08	0.3633	0.7369 (± 0.04)	61.11	38.62% - 79.69%	72.73	64.18% - 79.87%	2.241	*0.0012*

Cut-off: refers to the optimized threshold values for distinguishing between the compared groups, derived from the Receiver Operating Characteristics (ROC) curves. AUC (± SD): the area under the curve with its standard deviation (SD), measuring the overall test accuracy. Sensitivity (%): is the proportion of true positives identified, with its 95% Confidence Interval (C.I.), while Specificity (%) is the proportion of true negatives identified, with its respective C.I. Likelihood ratio: the ratio of the probability of a positive test result in true positives versus false positives. *P-values* represent statistical significance.

### 3.5 Immunodominance of *Pv*Vir14-derived peptides

**Figure 6:**
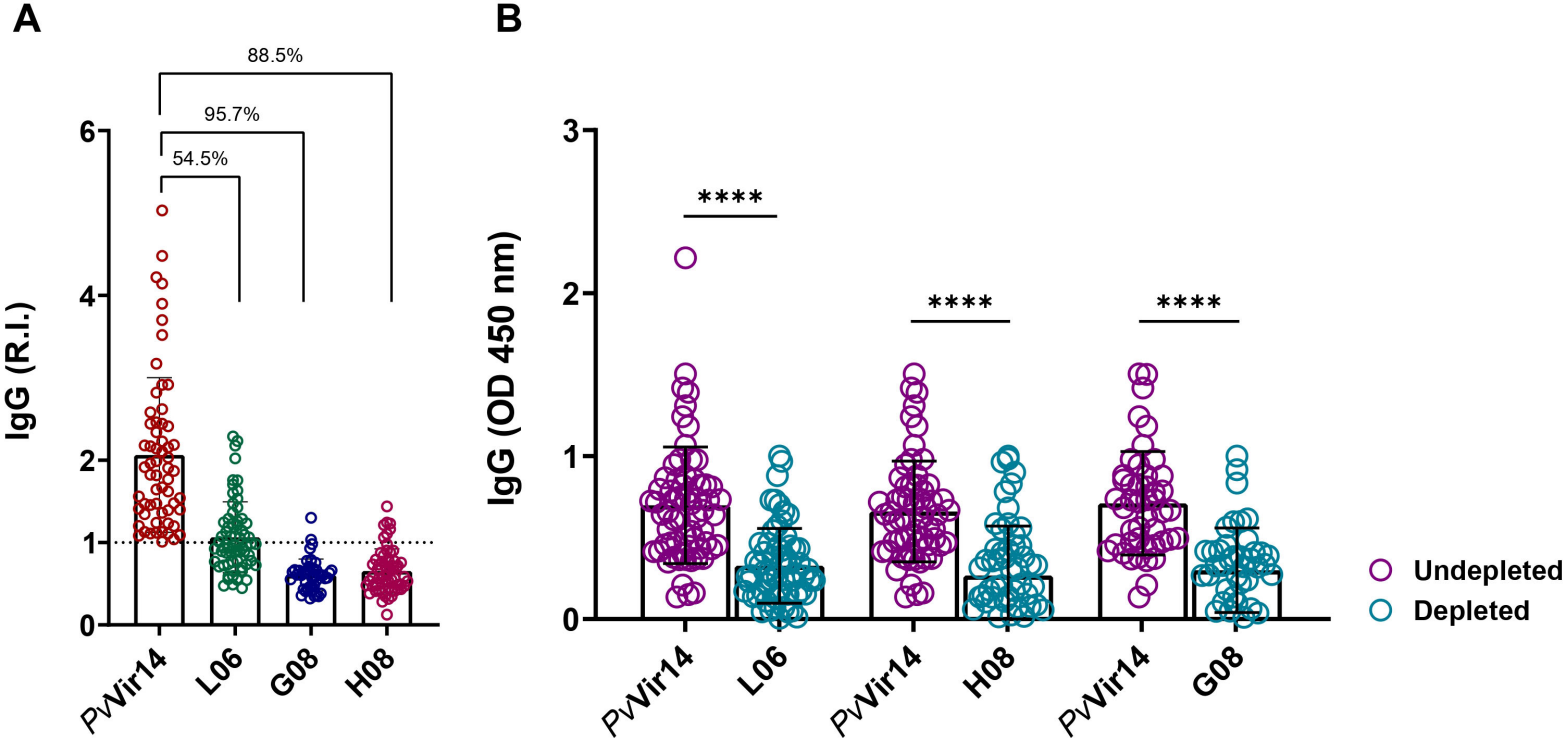
Immunodominance of PvVir14-derived peptides on the specific IgG reactivity. **(A)** Reduction on the anti-PvVir14 IgG reactivity frequency after depletion ELISA using the three selected peptides. **(B)** Reduction on the anti-PvVi14 IgG antibodies levels after depletion ELISA using the three selected peptides. The specific IgG levels are represented as optical density (OD). The values in “B” are expressed as mean ± standard deviation.

To analyze the influence of peptides over the *Pv*Vir14 recognition by specific IgG, a depletion ELISA was performed. The results indicated that the peptides L06, G08, and H08 exhibited immunodominance over the protein, as evidenced by a reduction in reactivity of 54.5%, 95.7%, and 88.5%, respectively (**Figure 6A**). This was further corroborated by the pronounced decline in IgG titres that recognized *Pv*Vir14 following depletion (**Figure 6B**). Specifically, prior to depletion, the mean optical density (OD) values for *Pv*Vir14 were 0.6980 (± 0.3577), 0.6600 (± 0.3095), and 0.7106 (± 0.3171), reflecting the baseline recognition of *Pv*Vir14 by IgG antibodies. After depletion using peptide L06, the mean OD decreased to 0.3270 (± 0.2294), while depletion with peptides H08 and G08 reduced the mean OD to 0.2681 (± 0.3035) and to 0.2995 (± 0.2597), respectively. These results demonstrate that while all three peptides contribute to *Pv*Vir14 recognition, the peptides H08 and G08 exhibit the highest level of immunodominance, significantly diminishing the protein recognition by specific IgG.

## 4 Discussion

Improving the diagnosis of *Plasmodium vivax* malaria is pivotal for disease control and eradication efforts. In endemic regions, a precise diagnosis is crucial to effectively disrupt the transmission cycle and ensure that patients receive adequate treatment to eliminate both blood and liver stages of the parasite, reducing the risk of relapse and further transmission (Baird et al., 2016; WHO, 2018).

Our study aimed to identify peptides derived from the *Pv*Vir14 protein (Fantin et al., 2022; Fantin et al., 2023) that are recognized by IgG antibodies in the sera of individuals with acute *P. vivax* malaria. Previous analyses of *Pv*Vir14 have shown that this protein does not share similarities with *P. falciparum* proteins or IgG cross-reactivity (Fantin et al., 2022; Fantin et al., 2023). The lack of cross-reactivity of antibodies to *Pv*Vir14 with *P. falciparum* underscores its potential as a highly specific biomarker for *P. vivax* infection.

Herein, the peptides H08, G08, and L06 displayed, individually, significantly higher reactivity suggesting that they may contain epitopes that are particularly effective in eliciting an IgG response in individuals from malaria-endemic areas. When the tripeptide was analyzed, the frequency of reactivity increased significantly, indicating a synergistic effect and underscoring their potential for *P. vivax* diagnostic purposes. It has previously been shown that combination of multiple antigens, either by using crude antigen extract or by combining specific proteins from *P. falciparum*, can improve the detection of specific IgG antibodies in the sera of malaria patients (Rouhani et al., 2015).

Although the frequency of IgM reactivity to *Pv*Vir14 and the peptides was lower in infected individuals, IgG reactivity to these four targets was significantly higher in this group, which is in accordance with previous analysis of *Pv*Vir14 immunological characteristic (Fantin et al., 2023). Previous research using the PvMSP10 and PvMSP8 proteins as biomarkers of infection exposure showed a good performance in detecting individuals with recent *P. vivax* infection (Villasis et al., 2021). More recently, analyses of the antigenic abilities of the *P. vivax* RBP2b derived fragments exhibited higher specific IgG antibody responses among individuals with active *P. vivax* malaria (Bourke et al., 2022). The use of distinct recombinant proteins, and peptides derived from many *P. vivax* proteins, such as *Pv*CSP, PvMSP1, *Pv*TRAP, and *Pv*AMA1 has demonstrated the capacity of naturally acquired antibodies from individuals residing in endemic *P. vivax* areas to efficiently recognize the antigens, representing a significant advancement in the identification of serological markers and novel vaccine targets (Bueno et al., 2011; Matos et al., 2019; Soares et al., 2020; Monteiro et al., 2021).

The detected reactivity of antibodies to specific *P. vivax* antigens has several important implications for understanding the immune response to malaria considering distinct transmission levels, acting as important serological markers for exposure (Rosado et al., 2021; Liu et al., 2022). In our study, IgG reactivity against the protein and the peptides was higher in individuals with multiple cases of malaria, suggesting that individuals with multiple exposures develop a higher immune response against the peptides, underscoring their potential as possible biomarkers for multiple infections. The naturally acquired immunity to malaria relies primarily on the exposure magnitude to the parasite, which includes relapse episodes by *P. vivax* hypnozoites (Mueller et al., 2013; Longley et al., 2016). Previous studies using distinct specific *P. vivax-*derived antigens have demonstrated an increased antibody response in individuals with a prior history of vivax malaria (Cerávolo et al., 2005; Morais et al., 2006; Yildiz Zeyrek et al., 2011; Cutts et al., 2014). In this sense, antigens that are strongly recognized by specific antibodies can identify levels of exposure in endemic areas for *P. vivax* malaria.

The variability in antibody reactivity among different populations, such as those in endemic versus non-endemic areas can provide valuable information about the different immune profiles in distinct populations. In this context, assessing the reactivity of the selected peptides in individuals from a non-endemic area in Brazil that experienced *P. vivax* malaria outbreak was crucial in identifying those different features. Individuals from non-endemic areas who have experienced symptomatic malaria once have been found to retain specific circulating anti-IgG and memory B cells even years after infection (Braga et al., 1998; Morais et al., 2005). In our study, the overall IgG reactivity frequency within individuals affected by the outbreak was lower than that observed for individuals living in endemic areas. These results suggest an interesting variation within the dynamics of the immune response between non- and endemic settings, which may be due to variations in exposure history and immune system priming.

By acknowledging the lack of similarities or cross-reactivity between *Pv*Vir14 and *P. falciparum* proteins (Fantin et al., 2022; Fantin et al., 2023) the diagnostic potential of the protein and its derived peptides was evaluated by analyzing their sensitivity and specificity. In the context of infected versus healthy individuals, *Pv*Vir14 demonstrated the highest diagnostic performance, highlighting its robust ability to distinguish infection status. Among the peptides, L06 achieved comparable performance, while G08 and H08 were shown to be slightly less consistent when compared to *Pv*Vir14 and L06 in this diagnostic scenario. For infected versus exposed individuals, *Pv*Vir14 again demonstrated superior diagnostic performance, while the derived peptides, however, showed more limited performance. Our findings propose that while L06, H08, and G08 are effective at identifying infected individuals, *Pv*Vir14 remains superior in differentiating infection status among exposed populations. Furthermore, the peptides L06, G08, and H08 represent major epitopes within *Pv*Vir14, as evidenced by their immunodominance, and act as driving a substantial portion of the IgG response to the protein.

The findings presented here have significant implications for understanding the immune response during *P. vivax* infection. These peptides, particularly L06, H08, and G08, show promising potential for incorporation into current diagnostic platforms such as ELISA or lateral flow assays. Given their ability to elicit strong IgG responses, they could serve as effective biomarkers for detecting *P. vivax* infection and the incorporation of these peptides into diagnostic tests could enhance the sensitivity and specificity of current assays, especially in resource-limited settings where rapid and reliable diagnostics are essential. However, there are challenges to consider when implementing these peptides in field settings. While peptide-based assays have high specificity, their sensitivity may vary depending on the population’s immune profile and the timing of infection, which could pose challenges for early-stage detection. Furthermore, validation of these assays in diverse field settings and different endemic regions is crucial for confirming their reliability and performance across varying transmission levels.

## Data Availability

The original contributions presented in the study are included in the article/**Supplementary Material**. Further inquiries can be directed to the corresponding author.
