# Supplementary material for: Immunogenicity of PvVir14-derived peptides to improve the serological diagnosis of Plasmodium vivax infection
Source: Front Cell Infect Microbiol. 2025 Mar 31;15:1484863. doi: 10.3389/fcimb.2025.1484863 (PMC11994686; doi:10.3389/fcimb.2025.1484863)
Supplement: Supplementary file 1 [file DataSheet1.pdf]

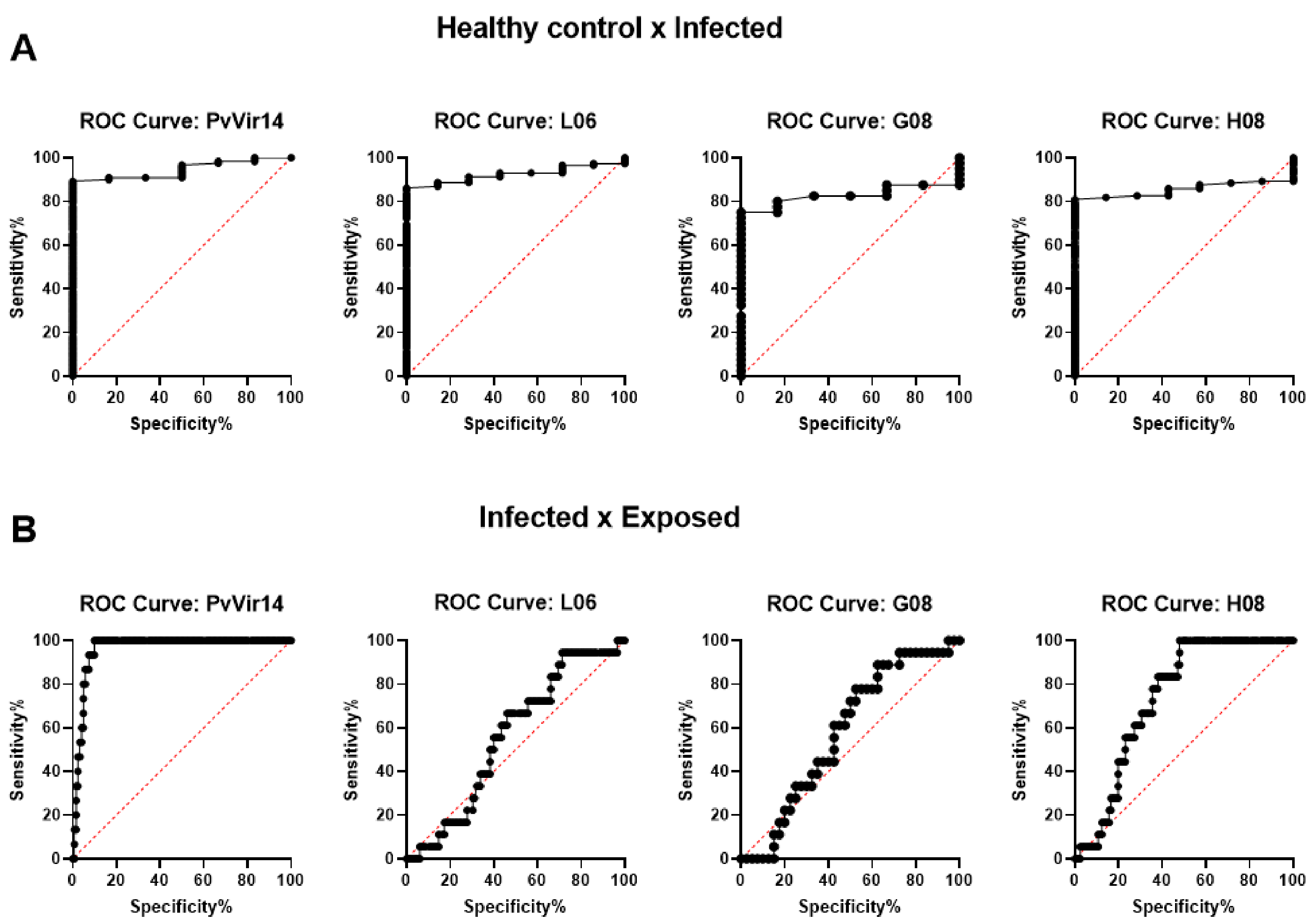

Fig. S1: Sensitivity and specificity assessment for PvVir14 and the three peptides for infected individuals from endemic areas. (A) Receiver Operating Characteristic (ROC) curves for sensitivity and specificity of the PvVir14 protein, and its derived peptides L06, G08, and H08, to discriminate between infected and healthy individuals, from endemic and non-endemic areas, respectively. (B) ROC curves for sensitivity and specificity of the PvVir14 protein, and its derived peptides L06, G08, and H08, to discriminate between infected and exposed individuals from endemic area.
